# Bioeconomic analysis of child-targeted subsidies for artemisinin combination therapies: a cost-effectiveness analysis

**DOI:** 10.1098/rsif.2014.1356

**Published:** 2015-06-06

**Authors:** Eili Y. Klein, David L. Smith, Justin M. Cohen, Ramanan Laxminarayan

**Affiliations:** 1 Center for Disease Dynamics, Economics and Policy, Washington, DC, USA; 2 Department of Emergency Medicine, Johns Hopkins University, Baltimore, MD, USA; 3 Department of Zoology, University of Oxford, Oxford, UK; 4 Sanaria Institute for Global Health & Tropical Medicine, Rockville, MD, USA; 5 Clinton Health Access Initiative, Boston, MA, USA; 6 Princeton Environmental Institute, Princeton University, Princeton, NJ, USA; 7 Public Health Foundation of India, New Delhi, India

**Keywords:** *Plasmodium falciparum*, Affordable Medicines Facility for malaria, child-targeted subsidy, anti-malarial drug resistance, The Global Fund

## Abstract

The Affordable Medicines Facility for malaria (AMFm) was conceived as a global market-based mechanism to increase access to effective malaria treatment and prolong effectiveness of artemisinin. Although results from a pilot implementation suggested that the subsidy was effective in increasing access to high-quality artemisinin combination therapies (ACTs), the Global Fund has converted AMFm into a country-driven mechanism whereby individual countries could choose to fund the subsidy from within their country envelopes. Because the initial costs of the subsidy in the pilot countries was higher than expected, countries are also exploring alternatives to a universal subsidy, such as subsidizing only child doses. We examined the incremental cost-effectiveness of a child-targeted policy using an age-structured bioeconomic model of malaria from the provider perspective. Because the vast majority of malaria deaths occur in children, targeting children could potentially improve the cost-effectiveness of the subsidy, though it would avert significantly fewer deaths. However, the benefits of a child-targeted subsidy (i.e. deaths averted) are eroded as leakage (i.e. older individuals taking young child-targeted doses) increases, with few of the benefits of a universal subsidy gained (i.e. reductions in overall prevalence). Although potentially more cost-effective, a child-targeted subsidy must contain measures to reduce the possibility of leakage.

## 1. Background

The use of artemisinin as a monotherapy, which expedites the evolution of malaria parasite resistance, and the difficulty of accessing effective antimalarial treatment in malaria-endemic countries are two major challenges to malaria control and elimination. The Affordable Medicines Facility—malaria (AMFm) was proposed as a novel market-based solution to address both problems [1,2]. AMFm had three components: pooled procurement of artemisinin combination therapies (ACTs) at a global level with pre-negotiated prices with manufacturers; complementary interventions in countries to educate patients and ensure that ACTs were used appropriately; and a subsidy for first-line buyers of ACTs, intended to lower the end-user price of ACTs in the private sector to a level that could displace artemisinin monotherapies (AMTs) and make ACTs affordable for most malaria patients. The proposed subsidy was intended to be global; however, to assess how effective a subsidy would be in expanding access to ACTs through reduced prices (at the expense of monotherapy), a pilot national-scale trial of AMFm was implemented starting in 2010 by the Global Fund to Fight AIDS, TB and Malaria in seven countries in Africa: Ghana, Kenya, Madagascar, Niger, Nigeria, Tanzania (including Zanzibar) and Uganda [3].

The objectives of AMFm were twofold: to reduce the use of artemisinin as a monotherapy, thereby prolonging the effectiveness of artemisinin; and to expand access to a safe, effective and affordable antimalarial through the private sector. The results of the pilot appeared successful in achieving these objectives [3]. Despite operational challenges, the market share of AMT declined in all countries where it was widely available, and the market share of quality-assured ACTs (ACTq) increased in the private sector in all the countries except Niger and Madagascar [3,4].

Although the subsidy significantly reduced ACT costs and expanded the market share of ACTs in the private sector, the cost of the programme was greater than estimated: demand for ACTs outstripped available funding, which necessitated prioritizing orders for public facilities and children, causing a significant drop in the fulfilment of orders by the private sector in the latter part of 2011. Although resource constraints are one hurdle to broader adoption of AMFm, there have been other concerns expressed about the programme including the risk of overtreatment, the need to encourage patients with non-malaria fevers to receive appropriate treatment as well as the widespread use of ACTs leading to resistance. Concerns have also been expressed about the likelihood of the subsidy being captured by middle-men and not reaching the end consumer, but this has been largely addressed by the AMFm evaluation, which found that lower prices do reach the end consumer [3].

Despite the success of the pilot, the Global Fund decided to convert AMFm into a country-driven mechanism whereby individual countries could choose to allocate funds from within their country envelopes to subsidize treatment in the private sector [5]. Several countries are actively pursuing the integration of private sector co-payments into their new funding model envelopes, and though the availability of funds for these programmes will only be clear once applications to the Global Fund's New Funding Model are completed (http://www.theglobalfund.org/en/fundingmodel), it is unlikely that funding envelopes will be sufficient to cover subsidies in addition to all of the other competing priorities of malaria programmes in all cases. The scarcity of subsidy resources constrains choices in dealing with malaria. In evaluating options, in addition to AMFm, countries must now choose between programmes for bednets, indoor-residual spraying, training of community health workers, scaling up diagnostic testing and provision of ACTs through the public sector. One potential cost-saving measure regarding AMFm is focusing on a subsidy for children, the subpopulation most likely to die from malaria.

Several factors make a subsidy targeted at children potentially attractive: (i) it would focus on those believed to be most at risk from serious complications; (ii) identifying the target population is simple; and (iii) differential packaging for young children would make it easy to separate payment. Despite these advantages, the Institute of Medicine committee that initially recommended a subsidy for ACTs ruled out a partial subsidy in its original deliberations, reasoning that it would be difficult to prevent ‘leakage’ (older individuals taking young children's doses) of a subsidy targeted at a particular age group [1]. Older consumers would have easy access to ACTs intended for young children and be likely to take advantage of two-tier pricing by purchasing young children's doses and either underdosing or ‘stacking’—purchasing multiple packs to make a full dose.

Other factors could impact the effectiveness of an age-targeted subsidy. For instance, a subsidy aimed at children would not reap the potential benefits of lowering malaria transmission by reducing prevalence as a universal subsidy would. Moreover, there would be a significant risk of underdosing, which could contribute to the development of resistance to ACTs, if adults chose to take the treatments intended for children. Also, a partial subsidy aimed at young children would not displace AMT use in older individuals or reduce the influx of counterfeit artemisinin derivatives [6,7].

In this paper, we present an age-structured bioeconomic model of malaria based on prior published models of malaria to evaluate the impact and cost-effectiveness of a subsidy targeted at just children, as well as reductions in the cost of the subsidy. We explore the effectiveness, cost and cost-effectiveness of the subsidy for varying levels of leakage and the likelihood of emergence of resistance because of underdosing.

## 2. Material and methods

The ultimate purpose of an antimalarial subsidy is to avert mortality and morbidity. Thus, the age distribution of mortality is an important consideration, since targeting the subsidy to those age groups with the highest risk would most cost-effectively reduce mortality, assuming binding budget constraints and no externalities. According to this logic, a subsidy targeted towards children under 5-year olds may be highly cost-effective, because children of this age bear the majority of the mortality burden (though the magnitude of this difference is in dispute [8,9]). Thus, our analysis took a provider perspective and we estimate the number of deaths that could be averted, the number of DALYs that could be averted, the estimated cost and the expected cost-effectiveness of a subsidy targeted at children under 5 for the seven pilot countries of AMFm: Ghana, Kenya, Madagascar, Niger, Nigeria, Tanzania (including Zanzibar) and Uganda. We examined a subsidy for artemisinin combination drugs sold in the private sector targeted at children under 5 as well as a universal subsidy. Both options were compared to a scenario of no subsidy. In addition, the incremental cost-effectiveness of going from a targeted subsidy to a universal subsidy was also estimated. The costs included were those for providing the subsidy per pack as well as insurance and freight, but excluded administration and management costs of a subsidy programme as well as the costs of supporting interventions such as training and communications.

Our analysis was performed based on data from the final report of the AMFm Independent Evaluation Team [4] as well as previously published estimates of fever incidence and treatment-seeking behaviours [10]. We used a 5-year time horizon with a 3% discount rate. Estimates are based on a new analysis of a previously published model of malaria transmission and immunity [11,12]. The model was modified slightly: acquisition of clinical immunity to malaria—reduced frequency and severity of clinical symptoms—was considered to be a function of both age and infection [13,14], and we assume that the population is structured into young children (less than 5) and the rest of the population, where young children are more likely to become clinically sick and die, *δ_X_*, and *X* is either young children (1) or everyone else (2). However, in keeping with the current understanding of malaria epidemiology, clinically immune individuals are still assumed to become infected and infectious [15–18]; however, they become sick and seek treatment at a lower rate [19,20]. We assumed that only a proportion of the population purchased drugs in the private sector (and thus benefited from the subsidy) but that this was slightly higher in young children using the formula defined in Cohen *et al.* [10].

In addition to age structure, we also modified the model to account for drug resistance by assuming that individuals can be infected by drug-sensitive parasites or parasites resistant to one or more drugs. The drugs were assumed to be (i) non-artemisinin drugs, such as chloroquine or sulfadoxine-pyrimethamine (denoted as NAT), which are not used in combination with artemisinin; (ii) AMT; (iii) a partner drug that is used with artemisinin-combination therapy but not used alone (PMT); and (iv) ACT. Thus, individuals can be infected with the wild-type, *I*_W*.X*_; singly resistant infections, *I*_NAT*.X*_, *I*_PMT*.X*_ and *I*_AMT*.X*_; doubly resistant infections, *I*_NAT.PMT.*X*_, *I*_NAT.AMT*.X*_ and *I*_ACT*.X*_; and triply resistant infections, *I*_NAT.ACT*.X*_.

The primary outcomes were deaths and DALYs averted. DALYs averted were calculated based on the calculation and disability weights in Mathers *et al.* [21]. Specifically, we assume that DALYs = YLL*_X_* + YLD_E*.X*_ + YLD_N*.X*_, where YLL is years of life lost, YLD_E_ is the disability weight for an uncomplicated episode, YLD_N_ is the disability weight for an episode with long-lasting neurological complications. YLL and YLD_N_ assume that incidence occurs on average at age 2 years and six months for children under 5 and age 29 for the rest of the population, while uncomplicated episodes are expected to last 7 days. DALYs were discounted at 3%. Severe malaria was assumed to occur in 1 and 0.5% of cases for children and the rest of the population, respectively, and 1% of severe malaria cases were assumed to result in long-term neurological complications [22–25].

### 2.1. Entomology

The dynamics of infection in the model follow the notation of Macdonald [26], as modified by Smith & McKenzie [27]. Vectorial capacity (*V*), the number of infectious bites by a mosquito over its lifetime, is given by the formula *V* = *ma*^2^e^−*gn*^/*g*, where *m* denotes the number of mosquitoes per human and *a* is the number of bites on humans per mosquito per day. The instantaneous death rate is *g* (e^−*g*^ is the probability of a mosquito surviving 1 day) and *n* is the number of days required for sporogony.

The daily entomological inoculation rate (EIR), the number of infectious bites per person per day, is calculated as the product of vectorial capacity and the fraction of mosquitoes that are infectious, *P*/(1 + *aP*/*g*), where *P* is the proportion of the bites on the infected human population that infect mosquitoes. Because we assume differing transmission efficiencies, *c_X_*, for young children and everyone else, *P* = *c*_1_*I*_1_ + *c*_2_*I*_2_, where *c*_1_ > *c*_2_ and *I*_i*.X*_ is the sum of all children under 5 and the rest of the population infected by either drug-sensitive or drug-resistant infections. The force of infection (*h*), is calculated as *b*EIR, where *b* is the fraction of bites on humans that produce a patent infection, and *h*_i_, or the force of infection for each parasite phenotype, is the contribution of each phenotype to the total force of infection [28].

### 2.2. Drug treatment

Young children and everyone else are assumed to develop clinical symptoms, *f_X_*, and seek treatment in the private sector, *υ_X_*, at different rates. Thus, *ρ*_1_ = *f*_1_*υ_1_* and *ρ*_2_ = *f*_2_*υ*_2_. In addition, a fraction of young children, on becoming infected, are assumed to develop clinical symptoms and treat with drugs purchased in the private sector immediately, *ξ*. Those who are treated with effective drugs are assumed to clear infection, but treatment with ineffectual drugs is assumed not to clear the parasite and increases the probability that a child will die. We further assume that a fraction of individuals do not adhere to their medication, which results in underdosing. A fraction of them are still assumed to effectively clear the parasite. After a bout of clinical illness that is effectively treated, individuals are assumed to remain refractory to infection, *R*_i_, for a period of time (*ψ*) because of the prophylactic effects of drug treatment. Because not all fevers are due to malaria, we assume that a fraction of susceptible individuals (*ω*) develop non-malarial fevers and treat at a similar rate as infected individuals. Treated susceptible individuals are then assumed to remain refractory for a period of time. Which drug an individual treats with is driven by demand for each drug, which is generated using a nested constant elasticity of substitution (CES) utility function, as discussed in Laxminarayan *et al.* [29]. Briefly, utility is maximized between general consumption, *C*, and a composite good of effective drug treatments, *E*, where *σ_u_* is the substitution elasticity between *C* and *E*. The drug composition is a CES function over consumption of the possible drugs weighted by their respective effectiveness, and *σ_a_* governs the degree of substitution between different drugs and thus the extent to which a subsidy that lowers the price of ACTq will displace the use of NAT drugs as well as AMTs and non-quality-assured ACTs (ACTn, for details, see [29] and appendix A). The effect of the subsidy on the price of ACTq in the private sector was estimated from the final report on the first phase of AMFm [4] which surveyed prices of drugs before and after the introduction of the subsidy. Because there was a range on both the pre- and post-subsidy prices, for each simulation we drew both from independent samples and calculated the expected reduction due to the subsidy as the difference.

### 2.3. Resistance

We assume that there is a small probability that when an individual takes a drug, *ρ*_i_, the treatment will lead to drug resistance, *d_X_*. Furthermore, we assume that resistance is encoded by a single mutation that affords complete resistance to drug treatment. Because clinically sick individuals have such high parasite densities relative to asymptomatic individuals [30], we assume that only clinically sick individuals who treat are likely to generate resistance *de novo* [31]. We also assume that the partner drug (PMT) has a lower rate of *de novo* resistance when used in an effective combination with artemisinin (*d*_PMT*.*A_ < *d*_PMT_).

Mutations encoding antimalarial drug resistance either affect the concentration of drug in the parasite or erythrocyte [32] or alter the mechanism of action of the drug in the parasite [33]. These changes, which provide a benefit in the presence of drugs, have also been shown to produce a biological fitness cost to the parasite [34,35]. To account for this effect, we assume that drug-resistant parasites have a faster clearance rate than drug-sensitive parasites. In addition, multidrug-resistant parasites are assumed to have an even greater cost of resistance. Thus, if natural clearance takes 1/*r* days, drug-sensitive parasites clear at rate *r*_W_ = *r*, single-resistant parasites clear at rate *r*_NAT_ = *r*_PMT_ = *r*_AMT_ = *r*_W_*π*, double-resistant parasites clear at rate *r*_NAT.PMT_ = *r*_NAT.AMT_ = *r*_ACT_ = *r*_W_*π*^2^ and triple-resistant parasites clear at rate *r*_NAT.ACT_ = *r*_W_*π^3^*, where *π* is the fitness cost of becoming resistant to an additional drug. No distinction is made between clearance rates for children less than 5 and everyone else.

**Figure 1.**
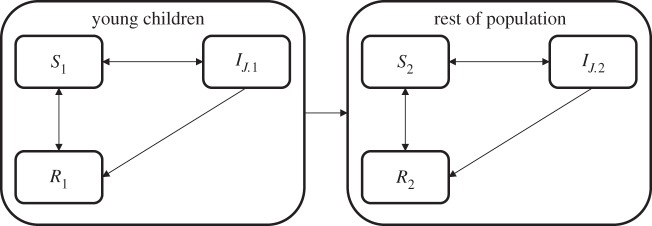
Schematic of transmission model. Diagram of the transmission model, where *J* refers to all possible infection states of wild-type and resistant infections. Susceptible individuals become infected and then either naturally clear and go back to being susceptible or treat with drugs and become prophylactically resistant to infection for a period before becoming susceptible again. Susceptible individuals who take drugs also become prophylactically resistant to infection. Young children progress to older age status after a period of time, regardless of disease status.

The dynamics of the system are described by a set of ordinary differential equations (appendix B and figure 1). The model is first run to equilibrium without drugs, we then run the model for 30 years with NAT drug therapy to get a baseline resistance level for the NAT therapies, and then run the simulations forward with artemisinin and partner drug therapy assuming that there is no resistance to these drugs at the outset.

### 2.4. Age-targeted subsidy

One potential consequence of a subsidy targeted solely at children is that older individuals will purchase young children's doses at subsidized rates for self-treatment (‘leakage’). This leakage of the subsidy would be expected to reduce the cost-effectiveness of the subsidy unless the benefits of reducing transmission outweigh the cost of treating individuals who are unlikely to die. We define leakage operationally in the model by assuming that a percentage of older individuals demand child doses. In this case a percentage of older individuals have a demand function that is determined by the subsidy (i.e. it is the same as the young child demand function). Correspondingly, we assume that increased leakage decreases the number of children less than 5 receiving subsidized treatments in a linear fashion. Thus, a percentage of young children have a demand function that is the non-subsidized demand function, which is the same as the older populations demand function. Furthermore, because young child doses contain less active ingredient than doses for older individuals, there is the possibility of underdosing, which has two effects: older individuals are less likely to effectively clear the parasite, increasing transmission; and inadequate doses expose the parasite to sublethal drug concentrations, increasing the probability of resistance. The harms of underdosing may be mitigated somewhat by ‘stacking’—the taking of multiple young child doses at one time, thus attaining a proper dose—though this comes at a cost of increased treatments subsidized. We assume that at baseline adherence to malaria therapy by all individuals is less than 100% [36], which reduces the rate that individuals clear the parasite and increases the rate of drug failure due to resistance. We further assume that a percentage of older individuals are underdosing, which has the same effect as lack of adherence, though individuals that stack are assumed to effectively clear the parasite. Thus, total demand under the age-targeted subsidy is the lowered children's demand plus excess demand from the rest of the population. Because we assume that leakage affects a percentage of the population, and the child population is less than half of the total population, this results in an increase in the total number of treatments demanded which is in relation to the population size.

### 2.5. Sensitivity analysis

We used a modified Monte Carlo method called Latin hypercube sampling (LHS) to assess the uncertainty of our results to the underlying assumptions about the parameters in each scenario [37,38]. LHS is significantly more efficient than simple random and fractional-stratified sampling designs at dealing with large numbers of input parameters because each input parameter is treated as a separate random variable [37]. Whereas a standard Monte Carlo simulation randomly selects each input parameter from within a probability distribution function, in LHS, each parameter distribution is stratified into equiprobable intervals and each interval is sampled exactly once, without replacement. An input vector is then generated, composed of the random samples of each of the input parameters for each simulation. LHS is efficient because each value of every parameter is used only once. The model may then be run *N* times to directly derive distribution functions for each of the outcome variables, and because of the probabilistic selection technique, the results can be interpreted within a statistical framework including calculation of partial rank correlation coefficients (PRCCs). PRCCs can help to determine the independent effects of each parameter on outcome variables, even when the parameters are correlated, and the relative importance of input variables in determining the imprecision of the result can be assessed by comparing PRCCs [37]. LHS has been used extensively to estimate uncertainty in epidemiological models similar to the model in this paper [39–44]. Parameters of the model are listed in table 1 and electronic supplementary material, table S1. Finally, for the incremental cost-effectiveness analysis, we used the bootstrap percentile method [45] to calculate confidence intervals.

**Table 1.** Parameters in model.

parameter	value	source**^a^**
entomological parameters		
human biting rate, *a*	0.3	
mosquito-to-human transmission efficiency, *b*	0.8	
human-to-mosquito transmission efficiency (children <5), *c*_1_	0.5	
human-to-mosquito transmission efficiency (population ≥5), *c*_2_	0.05	
mosquito death rate, *g*	0.1	
days to sporogony, *n*	10	
number of mosquitoes per human, *m*	varies^b^	[10]
drug treatment		
rate symptoms arise (children <5), *f*	varies^b^	[10]
rate symptoms arise (population ≥5), *f*	varies^b^	[10]
drug coverage rates (children <5), υ	varies^b^	[10]
drug coverage rates (population ≥5), υ	varies^b^	[10]
fraction of infections that are immediately clinical, treated and do not transmit (children <5), *ξ*	0.1	
refractory time period, *ψ* (d^−1^)	14	
susceptible individuals with non-malarial fever, *ω*	varies^b^	[10]
initial drug price	varies^b^	[4]
GDPPC	varies^b^	IMF
resistance		
rate of *de novo* resistance (NAT)	10^−1^	
rate of *de novo* resistance (ART)	10^−9^	
rate of *de novo* resistance (PMT)	10^−6^	
natural parasite clearance, *r* (d^−1^)	1/*b* × 165	[28]
fitness cost of resistance, *π*	triangular (6%, 2—10%)	
subsidy		
amount subsidy lowers ACT price	varies^b^	[4]
initial drug demand	varies^b^	[4]
years of initial ACT usage prior to subsidy	uniform (1–5 years)	
percentage increase in population ≥5 underdosing due to subsidy (child-targeted subsidy only)	triangular (50%, 25–75%)	
subsidy cost (children <5), US$	triangular (0.39, 0.32–0.46)	[46]
subsidy cost (population ≥5), US$	triangular (0.95, 0.61–1.30)	[46]
freight and insurance, US$	0.09	[46]
model parameters		
background mortality, *μ* (yr^−1^)	60	
disease-induced mortality (children <5), *δ_1_* (yr^−1^)	varies^b^	[10]
disease-induced mortality (population ≥5), *δ_2_* (yr^−1^)	varies^b^	[10]
immunity gain rate, *q*	5 yr^−1^	
population (children <5)	varies^b^	[10]
population (population ≥5)	varies^b^	[10]
discount rate	3%	
percentage of population adhering to therapy	triangular (82.5%, 65–100%)	[36]
percentage of underdosing/low adherence population effectively treated	triangular (50%, 25–75%)	

^a^Where source is not noted, the parameter is an estimate by the authors.

^b^Variation both by distribution and by country, values in electronic supplementary material, table S1.

## 3. Results

**Figure 2.**
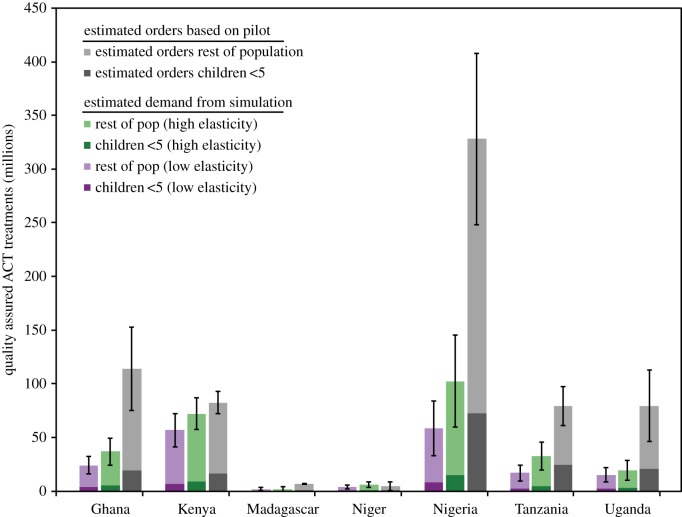
Estimated demand for quality-assured ACTs (ACTq) in the pilot countries with universal subsidy over 5-year time frame. Estimated demand for ACTq treatments by age-class for a universal subsidy. Estimates are for different demand elasticities, low (blue bars) and high (green bars), and for children under 5 (dark) and older individuals (light) treatments. The error bars are 1 s.d. of the mean of our sensitivity analysis for total ACTq demanded. These are compared to an extrapolated 5-year estimate of the total number of ACTq orders by the private-for-profit sector of each country based on data from the Global Fund for ACTq requested (not delivered) through the AMFm subsidy (grey bars) from 2011 to 2013 (see text for calculation) for child doses (dark) and all other doses (light). The error bars are 1 s.d. of the mean of our sensitivity analysis of ACTq requested.

As a comparator, we first estimated the demand for ACTs under the universal subsidy case, and compared the total demand broken down by age class against the total number of ACT doses ordered (not filled) from the Global Fund [46]. Figure 2 presents the mean and uncertainty of the LHS sensitivity analysis for low- and high-elasticity values. Because yearly summaries were only available for 4 years, and the subsidy did not start until partway through 2010, we averaged the total treatments ordered for the years 2011–2013 and multiplied by 5 to create an estimate of the total likely demand over 5 years with confidence intervals based on the variability in demand across years. Our estimates for annual ACT demand are significantly lower than the estimated number of orders reported by the Global Fund across elasticities and countries. The primary drivers in variation between country demand estimates were the initial fraction of ACTq use, the amount the price of ACT is reduced, population size and the share of the population using the private sector. Importantly, population size as a factor is more greatly affected by the size of the population 5 years and older, as they account for the majority of treatments. Underestimates of demand may be due to differences in heterogeneity in the transmission rate that we do not capture, underestimates of country-specific demand functions, or underestimates of overtreatment or private sector use. Alternatively, private for-profit entities may have been ordering more doses than demanded by consumers.

**Figure 3.**
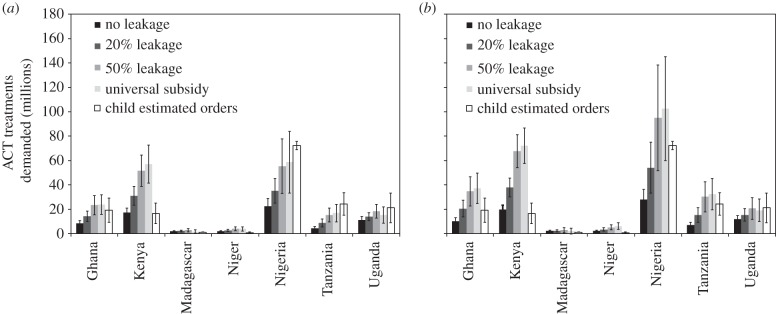
Estimated demand for quality-assured ACTs (ACTq) with child-targeted subsidy in pilot countries over 5-year time frame. Estimated demand for ACTq assuming that the subsidy targets only doses for children less than 5 with varying levels of leakage for two different demand elasticites, low (*a*) and high (*b*). Leakage assumes that older individuals are taking doses intended for children less than 5. Older individuals who take child doses are assumed to either ‘stack’ (take more than one child dose) or underdose. For those who underdose, only a proportion of population is assumed to adequately clear an infection, and the rest have an increase in the probability of resistance. The universal subsidy scenario is a subsidy for all ages. Because the universal subsidy reduces the prevalence rate, fewer children are infected and the total treatments for children is less than in the universal subsidy case. Bar heights are the mean and error bars are the uncertainty range (one standard deviation of the mean) of the sensitivity analysis. These are compared to an estimate of the total number of ACTq doses that would be demanded by the private-for-profit sector of each country based on data from the Global Fund for ACTq requested (not delivered) through the AMFm subsidy from 2011 to 2013 for child doses. The error bars are 1 s.d. of the mean of our sensitivity analysis of ACTq requested.

One important aspect of the analysis of projected ACT demand is that the majority of ACT treatments go to individuals over the age of 5 (the difference would be even greater if we used adult-equivalent dosing). This effect, combined with the fact that the majority of deaths may well occur in children, has led to the notion that the cost-effectiveness of the subsidy could be increased significantly by targeting child dosages. However, leakage could reduce the benefits of such a targeted proposal, we therefore estimated the effect of an age-targeted subsidy in each country assuming different levels of leakage to those 5 years and older. As leakage increased from 0% up to 50%, the number of ACT treatments demanded increased (figure 3). The age-targeted subsidy was also compared with a universal subsidy, and the number of child doses requested through the subsidy mechanism with 50% leakage is approximately equal to the total number requested under a universal subsidy because we are counting individual doses and not adult-equivalent, and a portion of those 5 years and older are assumed to be stacking.

Because we assume that deaths from malaria occur in individuals 5 years and older, we find, not surprisingly, that the number of deaths and DALYs averted is less than with a universal subsidy (table 2, electronic supplementary material, table S2). However, increasing leakage, even though it increases the number of treatments in the population as a whole, results in a reduced number of deaths averted. The reduction occurs because of the assumptions that leakage reduces children's access to subsidized doses and mortality in older individuals is lower. In addition, because leakage does not reach the full population, there is no indirect effect on mortality due to a reduction in the transmission rate. This contrasts with the universal subsidy case where both direct and indirect effects on mortality increase the averted number of deaths of children less than 5, though the indirect effect is not particularly significant (electronic supplementary material, figure S1). While death rates differ across countries, the effect of the subsidy on the number of deaths averted is not the same across countries because of differences in the estimated demand, population size and country-specific transmission rates. Because we used the pre- and post-ACT prices as reported in the final report on the pilot programme to estimate demand functions, the subsidy had only a limited effect in countries with a small change in the price of ACTs (e.g. Madagascar, which may have even seen an increase in the price of ACTs).

**Table 2.** Estimated number of deaths and DALYs averted from child subsidy versus universal subsidy over 5-year time frame, low elasticity.

country	no leakage	20% leakage	50% leakage	universal subsidy
deaths averted				
Ghana	970 (481–1459)	929 (424–1433)	843 (437–1249)	1,821 (985–2657)
Kenya	1134 (543–1724)	1135 (629–1641)	1127 (520–1734)	1935 (905–2965)
Madagascar	−37 (−240 to 165)	−85 (−265 to 96)	−37 (−271 to 198)	−127 (−559 to 305)
Niger	649 (280–1017)	608 (290–925)	463 (203–722)	1044 (415–1673)
Nigeria	14 200 (6073–22 328)	11 772 (6293–17 250)	9475 (3926–15 025)	20 445 (8756–32 133)
Tanzania	2078 (1110–3046)	1839 (975–2704)	1842 (916–2768)	3267 (1599–4934)
Uganda	428 (−36 to 892)	324 (−53 to 702)	303 (−71 to 676)	667 (−159 to 1494)
DALYs averted				
Ghana	69 796 (35 551–104 040)	66 247 (31 808–100 685)	60 599 (32 265–88 932)	131 957 (72 693–191 220)
Kenya	87 871 (43 170–132 572)	87 336 (50 783–123 889)	87 449 (42 420–132 478)	151 267 (75 596–226 938)
Madagascar	−2492 (−14 612 to 9628)	−5063 (−15 614 to 5488)	−1825 (−15 462 to 11 812)	−7502 (−32 282 to 17 278)
Niger	38 771 (17 252–60 291)	36 433 (18 023–54 842)	27 620 (12 479–42 760)	62 425 (24 644–100 207)
Nigeria	742 321 (319, 017–1 165 625)	611 832 (326 120–897 545)	480 603 (205 092–756 115)	1 039 205 (449 445–1 628 964)
Tanzania	131 715 (74 156–189 274)	119 876 (66 636–173 116)	117 802 (62 572–173 032)	207 780 (111 294–304 266)
Uganda	29 874 (−540 to 60 289)	23 272 (−3306 to 49 850)	21 450 (−4582 to 47 483)	48 626 (−11 916 to 109 169)

**Figure 4.**
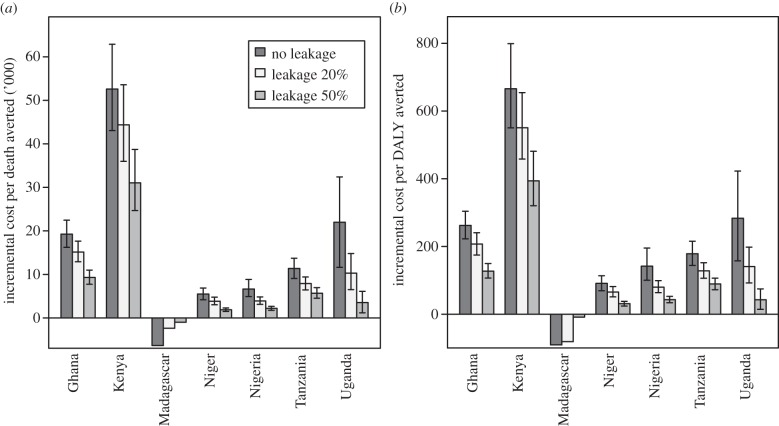
Incremental cost-effectiveness of universal subsidy compared with age-targeted subsidy, low elasticity. Although an untargeted subsidy is fairly cost-effective compared with the scenario of no subsidy, the incremental cost-effectiveness of a universal subsidy compared with the targeted subsidy, even with leakage, is quite large in most countries because of the paucity of malaria deaths in older age groups. This is true both for (*a*) deaths averted and (*b*) DALYs averted. Results are mean and 80% confidence interval for a bootstrap percentile method [45] of the sensitivity analysis results. No confidence intervals are shown for Madagascar because ICER values are negative.

Leakage also decreases the cost-effectiveness of the subsidy (table 3; electronic supplementary material, table S3), though a child-targeted subsidy costs significantly less than a universal subsidy, even with significant leakage. However, because of the relative paucity of malaria deaths in individuals 5 years and older, the incremental cost-effectiveness of moving from a child-targeted subsidy to a universal subsidy is high, unless a large amount of leakage is assumed (figure 4). We also found that over the time period of the study, resistance did not greatly impact the results, even when individuals 5 years and older were assumed to underdose more than under our baseline assumptions (electronic supplementary material, figure S2). Presumably a longer time frame would increase the possibility that such actions would affect resistance. Though the cost-effectiveness values calculated are similar in magnitude to prior estimates [12], great variation was observed between countries driven by differences in the death rates between countries, the effect of the subsidy on demand, and the cost of the subsidy (electronic supplementary material, table S4).

**Table 3.** Child-targeted subsidy cost and cost-effectiveness over 5-year time horizon, low elasticity.

country	no leakage	20% leakage	50% leakage	universal subsidy
subsidy cost (millions)				
Ghana	1.3 (0.7–2.0)	4.1 (2.2–6.0)	8.4 (5.0–11.9)	17.5 (9.6–25.3)
Kenya	2.6 (1.7–3.4)	9.1 (6.1–12.1)	19.0 (13.5–24.6)	43.6 (29.1–58.1)
Madagascar	0.0 (0.0–0.1)	0.1 (−0.1 to 0.3)	0.4 (−0.2 to 1.0)	0.4 (−0.4 to 1.2)
Niger	0.2 (0.1–0.3)	0.6 (0.3–0.9)	1.2 (0.6–1.7)	2.2 (1.0–3.5)
Nigeria	3.4 (1.5–5.4)	9.8 (5.7–14.0)	19.2 (9.8–28.7)	43.2 (18.0–68.4)
Tanzania	1.1 (0.6–1.6)	3.3 (1.8–4.8)	6.5 (3.7–9.2)	14.4 (7.8–20.9)
Uganda	0.4 (0.1–0.7)	1.6 (0.7–2.6)	3.8 (1.5–6.1)	4.9 (−0.4 to 10.3)
cost-effectiveness ($/death averted)				
Ghana	1425 (1135–1715)	4664 (3731–5598)	10 675 (8327–13 024)	9872 (7802–11 942)
Kenya	2952 (−151 to 6054)	9830 (3613–16 047)	23 590 (198–46 982)	29 459 (9781–49 138)
Madagascar	195 (−105 to 495)	1446 (−3255 to 6147)	8828 (−37 150 to 54 806)	1143 (−732 to 3018)
Niger	292 (208–376)	1035 (702–1367)	2787 (1963–3611)	2269 (1610–2928)
Nigeria	249 (192–306)	881 (679–1084)	2153 (1672–2634)	2168 (1603–2733)
Tanzania	557 (431–683)	1895 (1371–2419)	3779 (2714–4845)	4573 (3390–5757)
Uganda	774 (400–1149)	15 942 (−91 613 to 123 498)	19 062 (−37 694 to 75 818)	5858 (2875–8841)
cost-effectiveness ($/DALY averted)				
Ghana	19.55 (16.22–22.88)	64.43 (53.81–75.05)	146.04 (120.43–171.65)	134.91 (111.96–157.85)
Kenya	37.36 (2.29–72.44)	124.44 (48.73–200.15)	293.91 (40.65–547.17)	364.72 (133.67–595.78)
Madagascar	2.99 (−1.47 to 7.44)	19.40 (−59.07 to 97.86)	60.21 (−293.24 to 413.66)	18.13 (−10.45 to 46.70)
Niger	4.82 (3.57–6.07)	16.98 (12.18–21.79)	45.96 (33.77–58.15)	37.68 (28.13–47.23)
Nigeria	4.75 (3.67–5.82)	16.94 (13.11–20.77)	42.19 (33.37–51.02)	42.37 (32.45–52.29)
Tanzania	8.60 (7.07–10.14)	28.23 (22.65–33.82)	57.07 (45.35–68.78)	69.86 (56.02–83.71)
Uganda	10.82 (5.79–15.85)	25.83 (−217.49 to 269.16)	16.83 (−2221.66 to 2255.31)	79.69 (40.61–118.77)

## 4. Discussion

With the Global Fund's 2012 decision to permit recipient countries to include private sector co-payments as interventions supported by their funding envelopes [5], countries are now considering whether and how to include private sector subsidies as part of the package of interventions they will implement to fight malaria. These countries may choose to subsidize young child doses at a different rate or amount than doses for older individuals. This option is attractive because these treatments are cheaper and malaria is more likely to result in a young child's death than in individuals older than 5 years, but these benefits may be attenuated if targeting to children is unsuccessful. Our analysis has examined the potential benefits of a child-targeted subsidy in the countries where the pilot subsidy was implemented and found that it would likely be more cost-effective than a universal subsidy if there is only limited leakage in most countries, though it would likely avert fewer deaths. The benefit of a child-targeted policy was lower or less clear in Niger and especially Madagascar because the effect of the subsidy on ACT prices in both these countries relative to other countries was not as strong. The low impact of the subsidy in these countries was potentially due to differences in the structure of the private for-profit antimalarial sector, unfavourable politics and economic instability [3,4]. In the rest of the countries, the cost-effectiveness of the child-targeted subsidy was similar to other estimates for malaria-prevention programmes both measured as cost per death averted [12] and cost per DALY averted [47].

A major assumption of our model was that countries would be able to maintain the low price of high-quality ACTs; demand that is more elastic than we assumed could reduce the estimated benefits. For example, Nigeria has a high burden of malaria and had a relatively low penetration of ACTs during the pilot phase, raising the possibility that its future elasticity of demand may be significantly higher. This is a significant question, as under our high-elasticity scenario, though significantly more deaths would be averted, the cost of the subsidy in Nigeria would be more than double (electronic supplementary material, table S3). Higher than expected demand could lead to an inability to fully fund ACT demand (as happened in the first phase), which would keep the retail price higher and avert fewer deaths. In other words, an estimate of the effect of demand on the cost to subsidize ACTs should help countries determine how much funding to allocate, but less funding would necessarily result in a higher price in the shops and less effectiveness of the subsidy in averting deaths. Such problems are not a failing of a subsidy, but an indication that the demand for ACTs outstrips the supply.

Although a child-targeted subsidy may be more cost-effective if there is no leakage, there is a significant likelihood (as well as ample anecdotal evidence) that older individuals, including adults, will also take the doses that are targeted for young children. Because young children's doses would be fewer tablets or tablets formulated with less active ingredient, there is little to prevent older children and adults from taking them. Leakage would make fewer doses available for young children, and underdosing by older individuals would reduce the efficacy of the drug and increase the risk that drug resistance would emerge. The main benefit of a child-targeted subsidy (i.e. deaths averted) would thereby be attenuated, with few of the benefits of a universal subsidy (i.e. reductions in overall prevalence and thus indirectly mortality reduced) gained. A child-targeted subsidy must therefore include provisions for preventing leakage. Additional analysis should also be done to see where the most appropriate age cut-off lies. Our analysis assumed a child-age cut-off under the age of 5 years, as this is the most vulnerable population, but practicalities relating to development of pack-sizes might necessitate a slightly different age target, though we do not expect the qualitative results to vary significantly with a slightly older age cut-off.

As with all mathematical model-based exercises, the accuracy of our conclusions critically rests on our assumptions, including the validity of our malaria model and the model of demand for antimalarials. Although we have used the most comprehensive data from the preliminary assessment of the AMFm pilot [4], our estimates could significantly underestimate true demand because elasticities might be higher than expected. For example, in Nigeria, the subsidy increased the ACTq market share from 2% to only approximately 18%, and buyers in Nigeria ordered more than 100 million co-paid ACTs, or less than approximately 0.5 per person. Ghana, on the other hand, ordered 40 million co-paid ACTs, or approximately 1.6 per person. Our estimates of demand, however, were generally lower than the number of estimated ACTs that were ordered. Though not directly comparable, because we estimate the population seeking treatment in the private sector and the Global Fund orders are the number of drugs that first-line buyers tried to procure for the private sector, there are some potential reasons for this difference: (i) suppliers may have overestimated demand, or been trying to stockpile drugs given the knowledge that AMFm was time-limited, or they were trying to place as many orders as possible once order caps started to be implemented to limit procurement; (ii) suppliers may have been buying for the public sector through the private sector mechanism; (ii) procured drugs may have been flowing to other countries without the subsidy (i.e. a different form of leakage); and (iv) our estimates of demand are based on adult equivalent treatment doses (AETD) which may bias the demand function. Another issue may be our assumption that demand remains static through the simulation. Demand may continue to increase because of exogenous factors, such as learning-by-doing or educational outreach, that are not accounted for in our demand functions. Our substitution function also assumes that consumers value health interventions against a basket of consumer goods that remains unchanged over time, even though outside factors, such as the introduction of new goods, could alter their preferences [48]. In addition, a subsidy targeted at children may create a perception of scarcity in the market driving up the retail price of subsidized ACTs or could alter ACT stocking behaviour.

Another factor that could affect the results is our estimate of overtreatment, or the rate that individuals with fever not due to malaria are taking drugs. We assume that approximately 60% of feverish individuals without malaria parasites are treating, though we vary this amount over a large range. Though based on data, this rate could change with better information or with the introduction of rapid diagnostic tests [49]. An additional issue is that we focus solely on the private sector. Though the private sector is the main target of AMFm, ACT usage in the public sector may have indirect effects on the population as a whole due to lowered transmission. Lastly, our analysis assumes the country level as the unit of analysis, but significant variations in transmission and disease within countries could affect the cost-effectiveness at the country level.

## 5. Conclusion

Although AMFm as a global subsidy programme does not enjoy support from some donors, some countries are examining the possibility of providing ACTs through their private sector using funds from their country envelopes. This is a testament to the strength of the idea of leveraging the private sector to provide drug treatments. Given the budget constraints facing each country, a child-targeted subsidy may be both less costly and more cost-effective at averting deaths than a universal subsidy. However, leakage of the child-subsidized packs to older individuals would reduce the benefits (particularly with respect to averted mortality) and decrease the cost-effectiveness of the subsidy. Since countries are actively pursuing this option, strategies to mitigate this potential problem, such as packaging choices and educational campaigns, should be undertaken, along with studies to measure the extent of leakage and its consequences.

## Supplementary Material

Electronic Supplementary Material

## Appendix A. Demand functions

**A.1. Social planner utility function**

To generate drug demand functions, we adopted a nested, CES utility function defined following the notation of Laxminarayan *et al*. [29] over a period of duration , and *θ* is the discount rate:

where *k* is each possible drug (NAT, AMT, ACTns and ACT*q*), *α*_cost_ is the marginal disutility of infection and  is the probability of infection with a parasite resistant to drug *k*. The term in brackets is the utility from general consumption *C* and a composite good of effective drug treatments *E*. The responsiveness of aggregate drug use to changes in the price of ACTq due to the subsidy is governed by *σ_u_*, the elasticity of substitution between *C* and *E*.

**A.2. Elasticity of drug demand**

The drug composite, *E*, is a CES function over consumption of the four drugs weighted by their effectiveness:

where *e_k_* and *a_k_* measure the effectiveness of and the demand for each drug, and *σ*_s_ governs the degree of substitution between different drugs and therefore the extent to which an increase in demand for ACT*q* will displace use of the other drugs. *λ_k_* is a distribution parameter chosen to imply an observed initial drug mix and overall drug coverage rate.

**A.3. Demand functions**

As outlined in Laxminarayan *et al.* [29], with consumption constrained by a budget, demand functions for drug coverage for each drug *i* can be calculated as,

where *p* is the price of each drug, INC is the income of the household (assumed to be *per capita* gross domestic product) and  is an index of drug prices that accounts for drug effectiveness,

Thus, the proportion of individuals who demand each drug varies both with the price and effectiveness of each drug in relation to other drugs and with the overall price and effectiveness of drugs relative to other goods, though it does not vary with respect to malaria prevalence.

To calculate the effect of the subsidy on the demand for quality-assured ACTs, we used estimated market share and initial prices for each drug in the pilot countries as well as the effect of the full subsidy in changing the price of the drug. Initial market shares, initial prices and the final subsidized cost are shown in electronic supplementary material, table S1.

Electronic supplementary material, figure S3 shows an example of both the drug demand and the percentage demand for each drug, demonstrating that the CES predicts an increase in quality ACT use as well as an overall increase in demand, but that the subsidy must lower the price substantially to have an effect. The final drug demand of the full subsidy, as calculated by the CES utility function, approximates the estimated market share as described in the final evaluation report on phase 1 of AMFm [4].

## Appendix B. Epidemiological model

The following ordinary differential equations describe the mathematical model of malaria, where we assume that births (*B*) match deaths, that there is a background mortality rate (*μ*), and that children under 5 years become older at rate *q*.

**B.1. Equations for children**

**B.2. Equations for rest of population**
