# Supplementary material for: Bioeconomic analysis of child-targeted subsidies for artemisinin combination therapies: a cost-effectiveness analysis
Source: J R Soc Interface. 2015 Jun 6;12(107):20141356. doi: 10.1098/rsif.2014.1356 (PMC4590492; doi:10.1098/rsif.2014.1356)
Supplement: Electronic Supplementary Material [file rsif20141356supp1.doc]

**Supplementary Material for**

**Cost-effectiveness analysis of Targeting Children for Continuing Subsidies for ACTs Using a Bioeconomic Malaria Model**

Eili Y. Klein^1,2^, David L. Smith^3,4^, Justin M. Cohen^5^, and Ramanan Laxminarayan^1,6,7*^

^1^ Center for Disease Dynamics, Economics and Policy, Washington, DC, United States of America

^2^ Department of Emergency Medicine, Johns Hopkins University, Baltimore, Maryland, United States of America

^3^ Department of Zoology, University of Oxford, Oxford, United Kingdom

^4^ Sanaria Institute for Global Health & Tropical Medicine, Rockville, MD, USA.

^5^ Clinton Health Access Initiative, Boston, MA, USA.

^6^ Princeton Environmental Institute, Princeton University, United States of America

^7^ Public Health Foundation of India, New Delhi

* Corresponding Author

Ramanan Laxminarayan PhD, MPH

Director and Senior Fellow, Center for Disease Dynamics, Economics & Policy

1616 P St NW Ste 400, Washington DC 20036

Phone: 202.328.5085

Fax: 202.939.3460

Email: ramanan@cddep.org

| **Supplementary Table 1: Parameters varied in sensitivity analysis** | | | | | | | | | |
| --- | --- | --- | --- | --- | --- | --- | --- | --- | --- |
| **Parameter** | **Distribution** | **Country-specific values** | | | | | | | **Source** |
|  |  | **Ghana** | **Kenya** | **Madagascar** | **Niger** | **Nigeria** | **Tanzania** | **Uganda** |  |
| Number of mosquitoes per human, *m* | Uniform (±10%) | 0.87 | 0.31 | 0.42 | 0.41 | 0.58 | 0.36 | 0.67 |  |
| Rate symptoms arise (children <5), *f* | Triangular  (Low-High) | 0.26  (0.25–0.27) | 0.19  (0.18–0.20) | 0.19  (0.18–0.20) | 0.19  (0.18–0.20) | 0.21  (0.20–0.21) | 0.20  (0.19–0.21) | 0.28  (0.27–0.29) |  |
| Rate symptoms arise  (rest of pop), *f* | Triangular  (Low-High) | 0.15  (0.13–0.17) | 0.14  (0.12–0.17) | 0.11  (0.10–0.12) | 0.11  (0.09–0.12) | 0.13  (0.11–0.14) | 0.12  (0.10–0.13) | 0.17  (0.15–0.19) |  |
| Drug coverage rates (children <5), *υ* | Triangular  (Low-High) | 0.50  (0.41–0.59) | 0.34  (0.26–0.44) | 0.33  (0.24–0.42) | 0.43  (0.34–0.53) | 0.51  (0.44–0.58) | 0.40  (0.31–0.49) | 0.49  (0.39–0.58) |  |
| Drug coverage rates  (rest of pop), *υ* | Triangular  (Low-High) | 0.40 (0.36-0.43) | 0.26 (0.22-0.29) | 0.27 (0.21-0.33) | 0.35 (0.30-0.40) | 0.41 (0.40-0.42) | 0.31 (0.27-0.35) | 0.39 (0.35-0.42) |  |
| Overtreatment percent, *ω* | Triangular (±20%) | 63 | 84 | 65 | 72 | 60 | 77 | 57 |  |
| *Initial drug price ($US)* |  |  |  |  |  |  |  |  |  |
| NAT | Triangular  (Low-High) | 0.91  (0.34-3.08) | 1.18  (0.53-3.28) | 0.35  (0.35-0.47) | 0.39  (0.31-0.51) | 0.93  (0.52-1.49) | 1.41  (0.7-2.82) | 4.83  (1.16-7.25) | Table 2.3.1 |
| AMT | Triangular  (Low-High) | 5.14  (2.4-7.19) | 12.6  (7.88-22.68) | 22.45  (22.45-22.45) | 13.35  (6.43-22.52) | 4.09  (2.98-10.72) | 18.31  (6.52-25.32) | 16.72  (11.14-27.86) | Table 2.3.2 |
| ACTn | Triangular  (Low-High) | 3.77  (3.08-6.16) | 7.00  (4.59-12.25) | 5.61  (4.77-11.22) | 10.24  (8.05-16.61) | 4.47  (3.35-5.95) | 9.51  (6.73-14.6) | 4.64  (3.25-7.84) | Table 2.3.4 |
| ACTq | Triangular  (Low-High) | 3.42  (2.05-8.22) | 2.63  (1.31-6.3) | 0.14  (0.09-1.54) | 2.47  (2.06-4.11) | 4.47  (2.38-6.33) | 5.28  (1.41-8.45) | 2.79  (1.39-3.71) | Table 2.3.5 |
| Subsidized ACTq Price | Triangular  (Low-High) | 1.13  (0.94-1.88) | 0.58  (0.46-0.92) | 0.60  (0.34-1.37) | 1.19  (0.99-1.98) | 1.48  (0.89-2.36) | 0.94  (0.62-1.25) | 1.96  (1.17-2.82) | Table 2.3.5 |
| Percent Private Sector | Triangular (±20%) | 42.6 | 44.0 | 35.1 | 43.9 | 61.7 | 34.9 | 54.7 |  |
| GDPPC | Uniform (±20%) | 1,858 | 1,246 | 463 | 415 | 3,006 | 695 | 572 |  |
| *Initial drug demand (%)* |  |  |  |  |  |  |  |  |  |
| NAT | Triangular  (Low-High) | 54.8 (48.5-61) | 74.9 (63.8-86) | 92.9 (89.7-96.1) | 93.8 (90.5-97.2) | 84 (80.9-87) | 89.1 (80.4-97.9) | 69.5 (62.9-76.1) | Table 2.4.3 |
| AMT | Triangular  (Low-High) | 8.6 (4.2-13) | 2.3 (0.2-4.3) | 0.0 | 0.1 (0.0-0.3) | 8.7 (4.3-13.1) | 0.1 (0.0-0.2) | 0.6 (0.1-1) | Table 2.4.3 |
| ACTn | Triangular  (Low-High) | 30.2 (25-35.4) | 10.7 (4.1-17.3) | 0.2 (0.0-0.5) | 2.4 (1-3.8) | 5.1 (1.5-8.8) | 8.6 (1-16.2) | 24.9 (18.7-31) | Table 2.4.3 |
| ACTq | Triangular  (Low-High) | 6.5 (3.1-9.8) | 12.1 (6-18.2) | 6.8 (3.6-10.1) | 3.7 (1.2-6.2) | 2.2 (1.1-3.2) | 2.2 (1.1-3.3) | 5.1 (2.5-7.7) | Table 2.4.3 |
| Population (children <5) | Uniform (±10%) | 3,661,303 | 7,141,248 | 3,520,362 | 3,391,699 | 28,413,299 | 8,776,064 | 8,828,136 |  |
| **Supplementary Table 1: Parameters varied in sensitivity analysis, Cont.** | | | | | | | | | |
| **Parameter** | **Distribution** | **Country-specific values** | | | | | | | **Source** |
|  |  | **Ghana** | **Kenya** | **Madagascar** | **Niger** | **Nigeria** | **Tanzania** | **Uganda** |  |
| Population (rest of pop) | Uniform (±10%) | 26,474,503 | 45,309,706 | 22,754,023 | 17,468,346 | 173,890,985 | 50,352,091 | 47,678,138 |  |
| Disease Induced Mortality per 100,000 per year (children <5) | Triangular  (Low-High) | 289 (152-512) | 174 (82-304) | 55 (14-144) | 678 (206-1,302) | 938 (391-1,644) | 303 (125-566) | 262 (111-483) |  |
| Disease Induced Mortality per 100,000 per year (rest of pop) | Triangular  (Low-High) | 46 (25-67) | 33 (17-57) | 63 (29-122) | 43 (15-78) | 66 (39-118) | 35 (13-91) | 39 (23-66) |  |

| **Supplementary Table 2: Estimated number of deaths and DALYs averted from child subsidy vs. universal subsidy over five-year time frame, high elasticity** | | | | |
| --- | --- | --- | --- | --- |
|  | **Deaths Averted** | | | |
| **Country** | **No Leakage** | **20% Leakage** | **50% Leakage** | **Universal Subsidy** |
| Ghana | 1,564  (902-2,225) | 1,664  (909-2,419) | 1,482  (816-2,149) | 3,281  (1,940-4,621) |
| Kenya | 1,427  (698-2,156) | 1,499  (795-2,203) | 1,492  (734-2,250) | 2,484  (1,186-3,782) |
| Madagascar | -37  (-388-315) | -40  (-340-261) | -43  (-382-297) | -62  (-676-553) |
| Niger | 1,205  (555-1,855) | 1,083  (438-1,728) | 803  (335-1,270) | 1,886  (916-2,856) |
| Nigeria | 25,928  (11,433-40,422) | 24,114  (10,816-37,412) | 20,914  (7,540-34,289) | 42,016  (21,734-62,298) |
| Tanzania | 3,641  (1,977-5,305) | 3,638  (2,027-5,249) | 3,514  (1,856-5,171) | 6,514  (3,325-9,704) |
| Uganda | 701  (-89-1,491) | 571  (-23-1,164) | 475  (-92-1,042) | 1,224  (-2-2,449) |
|  | **DALYs Averted** | | | |
|  | **No Leakage** | **20% Leakage** | **50% Leakage** | **Universal Subsidy** |
| Ghana | 113,054  (67,323-158,786) | 118,692  (66,313-171,071) | 106,371  (59,766-152,976) | 230,943  (140,057-321,829) |
| Kenya | 109,172  (55,853-162,490) | 115,580  (63,166-167,993) | 116,769  (60,388-173,149) | 192,831  (98,171-287,491) |
| Madagascar | -2,601  (-23,257-18,055) | -2,269  (-20,133-15,595) | -2,398  (-21,685-16,889) | -3,162  (-38,978-32,654) |
| Niger | 72,129  (33,720-110,538) | 64,003  (26,733-101,272) | 47,584  (20,457-74,710) | 110,774  (54,732-166,816) |
| Nigeria | 1,352,055  (608,973-2,095,138) | 1,247,118  (561,154-1,933,081) | 1,058,352  (374,284-1,742,421) | 2,132,684  (1,103,898-3,161,471) |
| Tanzania | 235,848  (139,697-331,998) | 233,870  (136,039-331,701) | 222,992  (128,337-317,647) | 407,699  (233,024-582,375) |
| Uganda | 49,795  (-5,511-105,100) | 40,384  (-764-81,532) | 34,088  (-6,838-75,015) | 89,486  (1,441-177,530) |

| **Supplementary Table 3: Subsidy cost and cost-effectiveness over five-year time horizon, high elasticity** | | | | |
| --- | --- | --- | --- | --- |
|  | **Subsidy Cost (millions)** | | | |
| **Country** | **No Leakage** | **20% Leakage** | **50% Leakage** | **Universal Subsidy** |
| Ghana | 2.3  (1.3-3.2) | 6.9  (3.9-9.9) | 14.0  (8.5-19.6) | 30.5  (18.2-42.9) |
| Kenya | 3.8  (2.9-4.6) | 12.4  (9.6-15.2) | 26.6  (20.7-32.5) | 58.2  (42.6-73.8) |
| Madagascar | 0.0  (-0.1-0.2) | 0.2  (-0.2-0.5) | 0.5  (-0.4-1.3) | 0.7  (-0.8-2.2) |
| Niger | 0.3  (0.2-0.5) | 0.9  (0.4-1.4) | 1.8  (0.9-2.7) | 4.3  (1.9-6.7) |
| Nigeria | 6.3  (2.8-9.7) | 18.6  (9.2-28.0) | 38.4  (17.8-59.0) | 83.5  (44.1-122.9) |
| Tanzania | 2.3  (1.3-3.4) | 6.4  (3.6-9.2) | 13.4  (7.6-19.3) | 28.5  (15.8-41.2) |
| Uganda | 0.7  (0.0-1.4) | 2.3  (0.7-3.9) | 4.8  (1.2-8.5) | 8.7  (0.9-16.5) |
|  | **Cost Effectiveness ($/Death Averted)** | | | |
|  | **No Leakage** | **20% Leakage** | **50% Leakage** | **Universal Subsidy** |
| Ghana | 1,473  (1,189-1,757) | 4,277  (3,333-5,222) | 9,801  (8,000-11,601) | 9,575  (7,515-11,634) |
| Kenya | 3,950  (56-7,844) | 10,820  (2,970-18,671) | 23,970  (5,819-42,121) | 33,590  (4,444-62,737) |
| Madagascar | 155  (-127-436) | 994  (-1,516-3,503) | 2,653  (-2,465-7,771) | 1,150  (-541-2,841) |
| Niger | 300  (210-390) | 929  (635-1,224) | 2,461  (1,656-3,266) | 2,337  (1,687-2,987) |
| Nigeria | 257  (189-325) | 821  (619-1,022) | 2,016  (1,480-2,552) | 2,041  (1,555-2,528) |
| Tanzania | 671  (458-884) | 1,844  (1,330-2,359) | 4,042  (2,707-5,378) | 4,750  (2,937-6,564) |
| Uganda | 815  (426-1,204) | 5,243  (-3,738-14,225) | 19,395  (-39,050-77,839) | 5,920  (3,010-8,830) |
|  | **Cost Effectiveness ($/DALY Averted)** | | | |
|  | **No Leakage** | **20% Leakage** | **50% Leakage** | **Universal Subsidy** |
| Ghana | 20.13 (16.86-23.39) | 59.22 (48.32-70.11) | 135.19 (115.85-154.53) | 134.21 (111.50-156.92) |
| Kenya | 49.68 (3.31-96.06) | 137.28 (39.23-235.34) | 294.98 (97.48-492.48) | 419.12 (58.55-779.69) |
| Madagascar | 2.34 (-1.74-6.42) | -0.09 (-91.05-90.88) | 1,454.60 (-12,789.36-15,698.56) | 18.99 (-8.24-46.21) |
| Niger | 4.96 (3.59-6.32) | 15.51 (11.10-19.91) | 40.86 (29.29-52.43) | 39.24 (29.76-48.72) |
| Nigeria | 4.90 (3.64-6.15) | 15.79 (12.13-19.44) | 39.82 (29.76-49.87) | 40.13 (31.07-49.18) |
| Tanzania | 9.96 (7.66-12.26) | 28.13 (21.47-34.79) | 61.27 (46.32-76.22) | 71.86 (51.29-92.43) |
| Uganda | 11.34 (6.16-16.53) | 56.83 (-85.70-199.36) | 251.09 (-586.52-1,088.71) | 79.88 (43.18-116.58) |

**
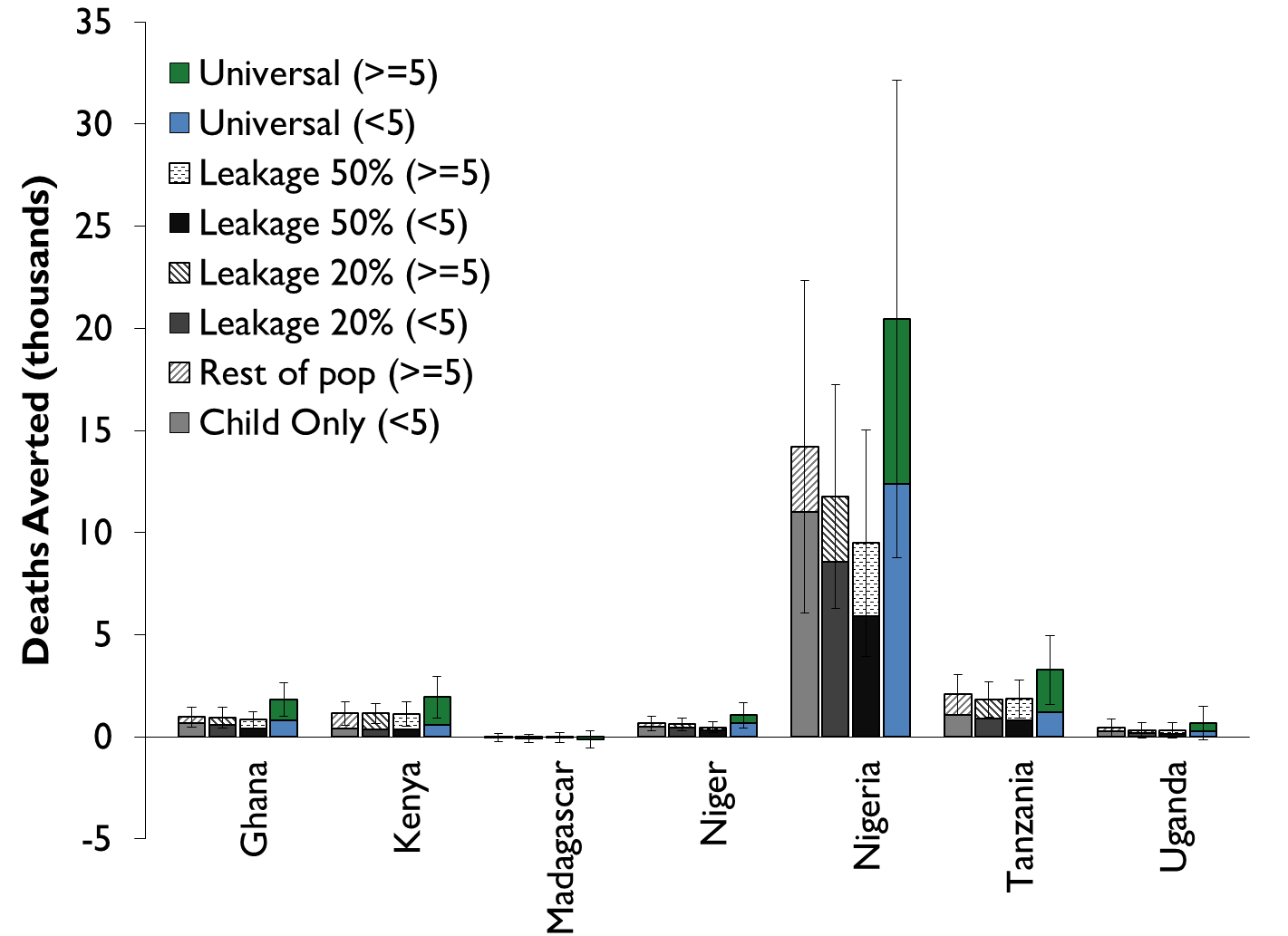
**

**Supplementary Figure 1: Estimated number of deaths averted from child subsidy vs. universal subsidy by age over five-year time frame, low elasticity**

The effect of a subsidy has both a direct effect on mortality and an indirect effect through a lower transmission rate. Increasing leakage doesn’t greatly affect the transmission rate, but a universal subsidy does decrease the transmission rate increasing the number of deaths of children under five that are averted, but only marginally. A universal subsidy is expected to also reduce deaths in older individuals.


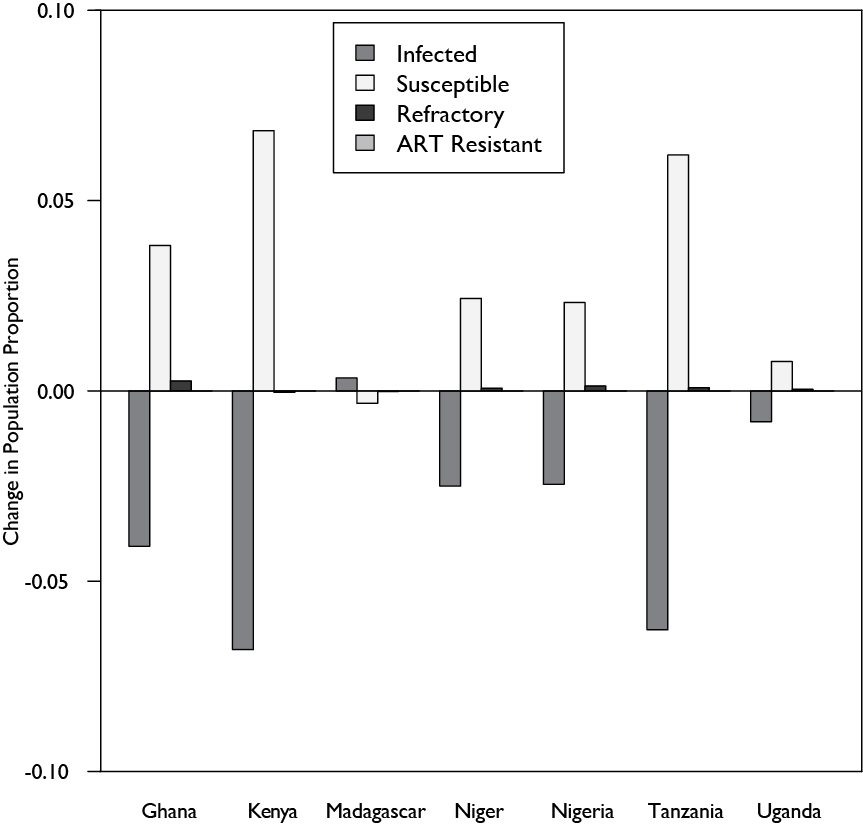


**Supplementary Figure 2: Change in estimated population proportions from before and after subsidy, low elasticity**

Introducing the subsidy generally increases the estimated amount of ACTq demanded by consumers in the model. This results in changes in the transmission rate which is reflected in a change in the estimated proportion of the population that is infected and susceptible at the endpoint of the model relative to the time before the subsidy is introduced. Refractory individuals also generally increase due to an increase in overall drug use. Finally, the proportion of individuals infected with artemisinin resistant parasites, which is a subset of the infected population, is not expected to greatly change over the course of the intervention because of the assumed low de novo rate of resistance and the relative short 5-year time frame. Results shown are for low elasticity simulation, high elasticity simulation is qualitatively the same.


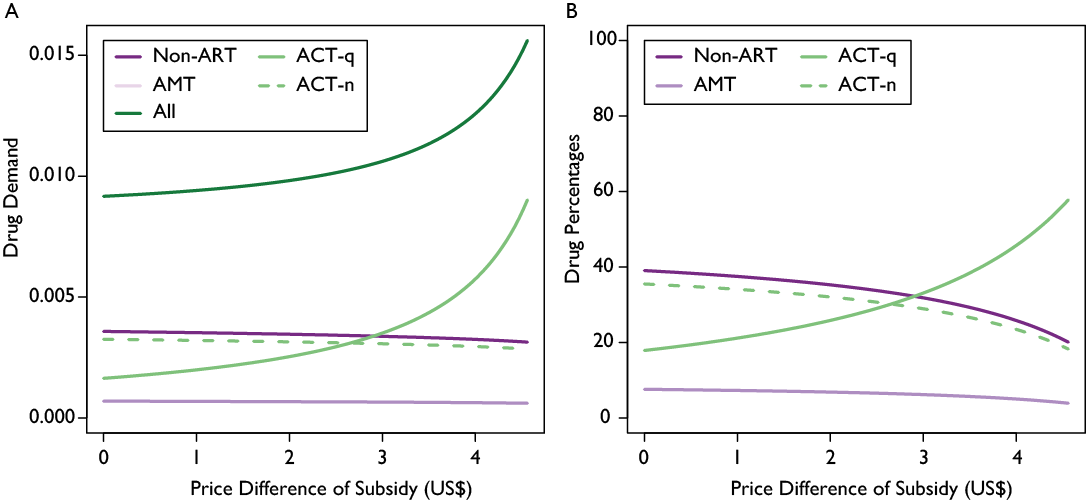


**Supplementary Figure 3: Example drug demand curves for different subsidy levels of quality-assured artemisinin combination therapies in pilot countries**

Drug demand is calculated by the CES utility function based on baseline prices and market share noted in final report on the first phase of AMFm with the x-axis noting the reduction in the cost of quality-assured ACTs. A) Displays the demand for each drug as calculated by the CES function as the amount the subsidy lowers the cost increases. B) Displays the percentage of each drug demanded as the amount the subsidy lowers the cost increases. Increasing the difference between the initial price prior to the subsidy and the subsidized price increases demand for quality-assured ACTs (ACT-q) and increases the percentage of the population using malaria drugs that uses quality-assured ACTs. The closer the subsidized price gets to the cost of non-artemisinin drugs (Non-ART), the greater the increase in demand for ACT-q, and the lower the demand for Non-Art, artemisinin monotherapy (AMT) and non-quality-assured artemisinin combination therapies (ACT-n). Different elasticities would also affect the change in demand. In the example above we assume a low elasticity.

| **Supplementary Table 4: Partial rank correlation coefficients for cost effectiveness, low elasticity** | |
| --- | --- |
| Disease-induced mortality (population ≥5) | -0.39*** |
| Disease-induced mortality (children <5) | -0.28*** |
| Reduction in ACTq | -0.25*** |
| ACTn initial drug demand | 0.25*** |
| Subsidy cost (population ≥5) | 0.24*** |
| CQ initial drug demand | -0.12*** |
| AMT initial drug demand | 0.11*** |
| Number of mosquitoes per human | -0.09** |
| Fitness cost of resistance | 0.09*** |
| ACTq Initial Price | -0.09** |
| Overtreatment percent | 0.08** |
| Population (population ≥5) | -0.07* |
| CQ Initial Price | 0.07* |
| Drug coverage rates (population ≥5) | 0.06 |
| Percent private sector (children <5) | 0.06 |
| AMT Initial Price | -0.05 |
| Rate symptoms arise (population ≥5) | 0.05 |
| ACTq initial drug demand | 0.05 |
| Drug coverage rates (children <5) | -0.05 |
| ACTn Initial Price | 0.05 |
| Rate symptoms arise (children<5) | -0.04 |
| Effective treatment of underdosing | 0.03 |
| Subsidy cost (children <5) | 0.02 |
| Percent underdosing | -0.01 |
| Percent private sector (population ≥5) | 0.01 |
| GDPPC | 0.00 |
| (***) Significant at the 1% level; (**) Significant at the 5% level; (*) Significant at the 10% level | |

**References**

[1] Cohen, J.M., Woolsey, A.M., Sabot, O.J., Gething, P.W., Tatem, A.J. & Moonen, B. 2012 Optimizing Investments in Malaria Treatment and Diagnosis. *Science* **338**, 612-614. (doi:10.1126/science.1229045).

[2] AMFm Independent Evaluation Team. 2012 *Independent Evaluation of Phase 1 of the Affordable Medicines Facility - malaria (AMFm), Multi-Country Independent Evaluation Report: Final Report*. Calverton, Maryland and London, ICF International and London School of Hygiene and Tropical Medicine.

[3] World Development Indicators. 2014 Washington, DC: World Bank. doi:10.1596/978- 1-4648-0163-1. License: Creative Commons Attribution CC BY 3.0 IGO.

[4] Murray, C.J.L., Rosenfeld, L.C., Lim, S.S., Andrews, K.G., Foreman, K.J., Haring, D., Fullman, N., Naghavi, M., Lozano, R. & Lopez, A.D. 2012 Global malaria mortality between 1980 and 2010: a systematic analysis. *The Lancet* **379**, 413-431.
